# The Utilization of Medical Devices by Traditional Korean Medicine Doctors Investigated through Traditional Korean Medicine Clinical Studies

**DOI:** 10.1155/2018/3987019

**Published:** 2018-08-09

**Authors:** Soo-Hyun Sung, Hee-Ju Sim, Eu-Gene Kim, Angela Dongmin Sung, Jung-Youn Park, Byung-Cheul Shin, Min-Jung Park, Chang Hyun Han, Jang-Kyung Park

**Affiliations:** ^1^Department of Pathology, College of Korean Medicine, Dae-gu Haany University, Daegu 38610, Republic of Korea; ^2^Department of Healthcare, Graduate School of Business, Ewha Womans University, Seoul 03760, Republic of Korea; ^3^Department of Food and Nutrition, College of Natural Sciences, Inha University, Incheon 22212, Republic of Korea; ^4^Department of Preventive Medicine, College of Korean Medicine, Sangji University, Wonju 26339, Republic of Korea; ^5^Department of Social Welfare, Sungkyunkwan University, Seoul 03063, Republic of Korea; ^6^Division of Clinical Medicine, School of Korean Medicine, Pusan National University, Yangsan 50612, Republic of Korea; ^7^Graduate School of Public Health, Seoul National University, Seoul 08826, Republic of Korea; ^8^Clinical Research Division, Korea Institute of Oriental Medicine, Daejeon 34054, Republic of Korea; ^9^Department of Obstetrics and Gynecology, College of Korean Medicine, Sangji University, Wonju 26339, Republic of Korea

## Abstract

**Objective:**

The purpose of this study was to investigate the current status of modern medical devices utilized in diagnosis and treatment in traditional Korean medicine (TKM).

**Methods:**

We searched the following six Korean electronic databases to collect TKM clinical studies that were published in a five-year period (January 2012 to December 2016). Clinical studies of TKM when medical devices were used for diagnosis or treatment were investigated.

**Results:**

The search generated a total of 3,735 articles, and 1,328 of these were considered to be clinical studies. Of a total of 1,328 clinical studies of TKM, 774 articles (58.3%) used medical devices for diagnosis or treatment, and 554 articles (41.7%) did not use medical devices for diagnosis or treatment. The three most used diagnostic devices were as follows: MRI scanners, which were used in 194 (20.6%) studies; X-ray machines, which were used in 172 studies (18.3%); and CT scanners, which were used in 139 studies (14.8%). The three most used treatment devices were electroacupuncture equipment (20.3%), transcutaneous electrical nerve stimulation (TENS) equipment (18.4%), and interferential current therapy (ICT) equipment (16.4%).

**Conclusions:**

This study suggests that TKM doctors use diagnostic information derived from modern medical devices clinically. It is therefore necessary to institutionalize considering changes to the medical acts of traditional medicine (TM) doctors. Additionally, this information can be utilized as a reference for developing TM policy and education.

## 1. Introduction

A medical device is an instrument, machine, device, material, or any other similar product used alone or in combination in humans or animals, as specified in the following: a product used for the purpose of diagnosing, curing, alleviating, treating, or preventing a disease; a product utilized for the purpose of diagnosing, curing, alleviating, or correcting an injury or impairment; a product used for testing, replacing, or transforming a structure or function [1]. According to the World Medical Market Report, the global medical device market will grow at an average annual rate of 4.8% from 2010 to 2014, rising to an annual average of 6.1% from 2015 to 2020 due to an increasing trend in aging and awareness [2]. It is estimated that the market size will grow to approximately $ 435.8 billion by 2020 [2]. The size of the medical device market in Korea also maintained a high annual growth rate of 10.4% from 2011 to 2015, and the production of medical devices reached $ 4.5 billion in 2015 [3].

In the past, doctors showed the medical behaviors of using stethoscope; recently, they presented the behaviors of utilizing modern medical devices (e.g., magnetic resonance imaging (MRI) scanners, computed tomography (CT) scanners, and medical laser) for diagnosing and treating patients [4, 5]. The reason why doctors have adopted modern medical devices is to gain a technical advantage and enhance self-determination and professional recognition [6].

Traditional tools such as acupuncture, herbal medicine, and cupping therapy have been used by traditional Korean medicine (TKM) doctors for a long time [7, 8]. Recently, TKM doctors have used medical devices such as electroacupuncture devices, electropulse graph, and distal arterial pulse wave analyzers, in combination with modern technology, for treatment [9, 10]. However, certain medical devices such as X-ray machines, MRI scanners, and CT scanners are not legalized for use by TKM doctors [11, 12].

China and Taiwan, similar to Korea, have a dual medical system that combines traditional medicine (TM) with conventional medicine (CM), and TM is included in the national system to provide medical service for nations [13]. In China, traditional Chinese medicine (TCM) doctors prescribe conventional medications, use modern medical devices, and perform surgeries, similar to licensed medical doctors [7, 14]. In Taiwan, only traditional treatments (e.g., acupuncture, moxibustion, and herbal medicine) were available to TM doctors, but recently complete blood counts, urinalysis, fecal analysis, and radiography have been allowed [7, 15]. Thus, the practice of TM doctors varies according to national systems and policies.

To our knowledge, the review on the use of medical devices by TKM doctors has not been published. The aim of this study was to investigate the current status of the medical behavior of TKM doctors by analyzing the data from clinical studies of TKM journals and to provide a reference for establishing TM policies and systems in countries that have a TM system

## 2. Methods

### 2.1. Data Sources and Searches

In April 2017, we searched the following six Korean electronic databases to collect TKM clinical studies that were published recent five years (January 2012 to December 2016); Korea Institute of Science and Technology Information, Korean traditional knowledge portal, KoreaMed, OASIS, RISS, and KISS. Clinical trials of TKM indexed in non-Korea databases such as PUBMED, EMBASE, or MEDLINE were not considered. The search keywords were as follows: “oriental medicine OR traditional medicine OR traditional Korean medicine OR complementary and alternative medicine” AND “clinical studies OR clinical trial OR case studies OR case report OR case series OR case controlled trial OR randomized controlled trial”.

We did not limit study languages, but we excluded studies that did not have a paper format (e.g., abstract-only articles and conference presentations). The studies of master and doctoral degrees were not considered for inclusion.

### 2.2. Study Selection

We included only clinical studies of TKM. Reviews, surveys, qualitative studies, and experimental studies were excluded. Cohort studies or clinical studies in which participants were not provided any type of treatment were also excluded. Three authors (H. J. Sim, E. G. Kim, and A. D. Sung) independently screened and selected papers that met the study criteria. Disagreements were resolved by discussion with two authors (S. H. Sung and J. K. Park) to arrive at a consensus

### 2.3. Definition of Medical Device

This study classified medical devices into two categories: diagnostic devices and treatment devices. Diagnostic devices include medical imaging equipment (e.g., X-ray machines, MRI scanners, CT scanners, and ultrasound scanners), blood testing equipment, and infrared thermographic equipment used to examine the human body. Treatment devices include equipment for electric needle treatment, equipment for electric stimulus treatment, and laser treatment devices. However, equipment such as needles, lancets, and cups for cupping were excluded from the study as they were not considered to be machines.

### 2.4. Data Extraction

In TKM clinical studies where medical devices were used for treatment or diagnosis, we gathered information regarding the medical device. The collected information was classified into diagnostic devices and treatment devices. Furthermore, in order to investigate the current status of medical device use per year and the type of diseases, relevant information was gathered and further analyzed. Two independent reviewers (J. Y. Park and C. H. Han) extracted data using a form designed in the review. Discrepancies were resolved by discussions with two other reviewers (S. H. Sung and B. C. Shin).

When there were multiple diseases, the more serious disease was put first, and studies that focused on healthy people or case studies that treated more than 2 people with multiple diseases were excluded.

## 3. Results

### 3.1. Study Selection and Description

**Figure 1:**
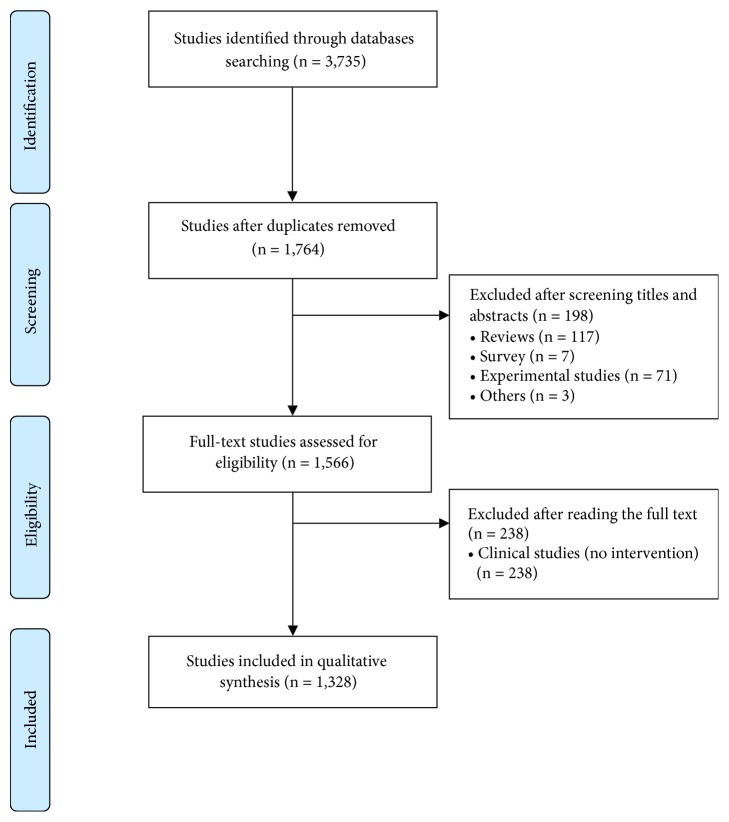
Flowchart of the study selection process.

The search generated a total of 3,735 articles, of which 1,328 were considered to be TKM clinical studies. Four hundred and thirty-six studies were excluded for the following reasons: 117 studies were reviews, seven studies were surveys, 71 studies were experimental studies, 238 studies were clinical studies without interventions, and three studies were abstract-only articles or conference presentations (Figure 1).

### 3.2. Utilization of Medical Devices Classified by Type

#### 3.2.1. Utilization of Medical Devices

From a total of 1,328 clinical studies of TKM, 774 articles (58.3%) used medical devices for diagnosis or treatment, and 554 articles (41.7%) did not use medical devices for diagnosis or treatment.

Medical devices were used in TKM clinical studies a total of 1,345 times from 2012 to 2016; diagnostic devices were used 942 times (70%), while treatment devices were used 403 times (30%) (Table 1).

#### 3.2.2. Utilization of Diagnostic Devices

Diagnostic devices were used 942 times in the TKM clinical studies. The 10 most used diagnostic devices are illustrated in Table 1. MRI scanners were used in 194 (20.6%) studies; X-ray machines were used in 172 studies (18.3%); CT scanners were used in 139 studies (14.8%); electrocardiography (EKG) equipment was used in 80 studies (8.5%); ultrasound scanners were used in 57 studies (6.1%); digital infrared thermal imaging (DITI) equipment was used in 43 studies (4.6%); heart rate variability (HRV) measurement equipment was used in 41 studies (4.4%); body composition analyzers were used in 28 studies (3.0%); electromyography (EMG) equipment was used in 21 studies (2.2%); and endoscopes in 19 studies (2.0%).

#### 3.2.3. Utilization of Treatment Devices

In the 1,328 clinical studies investigated, medical devices were used for treatment 403 times. The 10 most used treatment devices are listed in Table 1. Electroacupuncture devices were used in 82 studies (20.3%); transcutaneous electrical nerve stimulation (TENS) equipment was used in 74 studies (18.4%); interferential current therapy (ICT) equipment was used in 66 studies (16.4%); ultrasound and infrared (IR) devices were used in 23 studies (5.7%); microwave therapy (M/W) equipment were used in 19 studies (4.7%); electrical stimulation treatment (EST) equipment was used in 12 studies (3.0%); lasers and silver spike points (SSPs) were used in 11 studies (2.7%); and electromagnetic stimulation therapy equipment was used in 6 studies (1.5%).

### 3.3. Utilization of Medical Devices per Year

#### 3.3.1. Utilization of Medical Devices per Year

**Figure 2:**
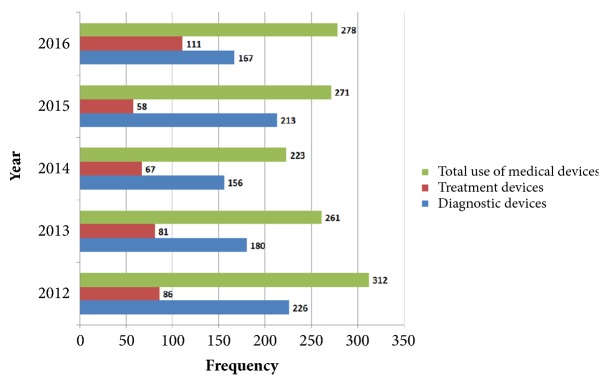
Utilization of medical devices in Korea from 2012 to 2016 by publication year.

The use of medical devices decreased from 2012 to 2014 and increased by 2016. Medical devices were used the most (312 times) in 2012. The utilization of diagnostic devices revealed a decreasing trend overall. The use of treatment devices declined from 2012 to 2015, and a steep increase occurred in 2016 (Figure 2).

#### 3.3.2. Utilization of Diagnostic Devices per Year

**Figure 3:**
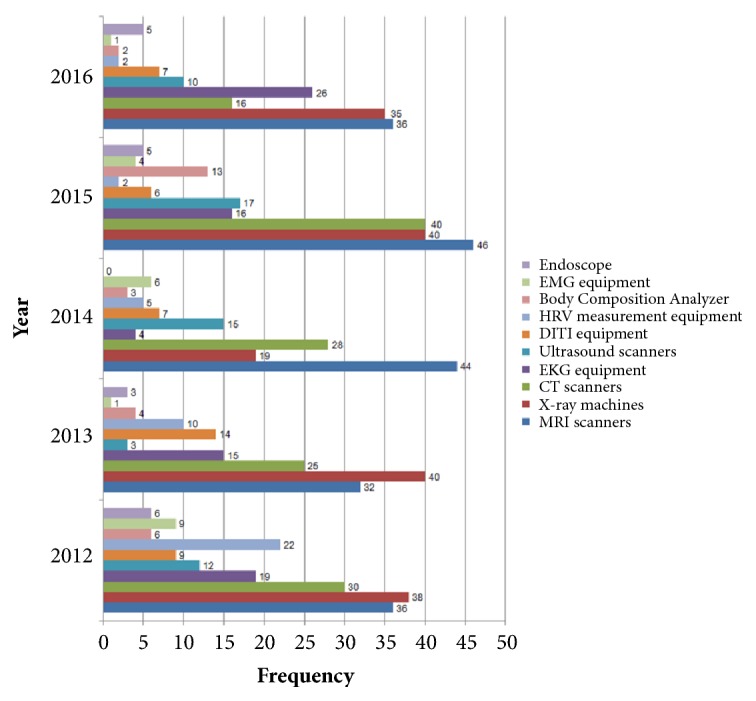
Study trend of diagnostic devices reported in clinical studies of TKM per year. MRI: magnetic resonance imaging; CT: computed tomography; EKG: electrocardiography; DITI: digital infrared thermal imaging; HRV: heart rate variability; EMG: electromyography.

Investigation of the 10 most used diagnostic medical devices per year in TKM clinical studies over the past 5 years revealed that MRI scanners, X-ray machines, and CT scanners were the most used medical devices from 2012 to 2016. MRI scanners were used a minimum of 32 and a maximum of 46 times every year. X-ray machines were used from 19 to 40 times, and CT scanners were used from 16 to 40 times. Electrocardiography use showed a decreasing trend from 2012. However, an increasing trend was demonstrated from 2014. Heart rate variability machines were not used much in 2015 and 2016, whereas they were used in 22 studies in 2012 (Figure 3).

#### 3.3.3. Utilization of Treatment Devices per Year

**Figure 4:**
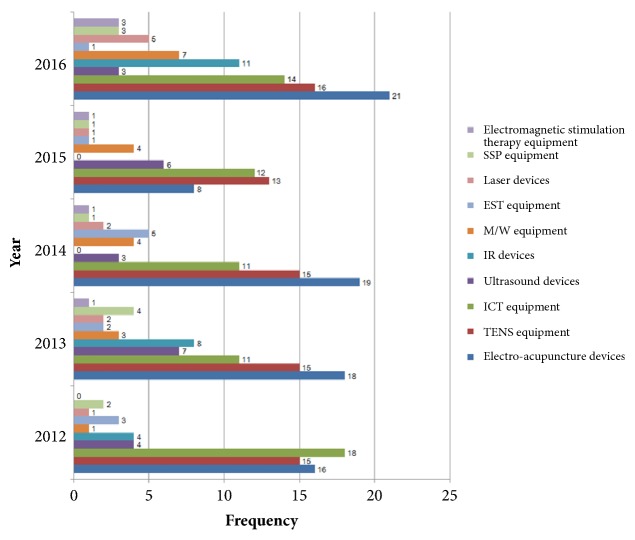
Study trend of treatment devices reported in clinical studies of TKM per year. TENS: transcutaneous electrical nerve stimulation; ICT: interferential current therapy; IR: infrared rays; M/W: microwave therapy; EST: electrical stimulation therapy; SSP: silver spike point.

Assessment of the 10 most used treatment devices that were utilized in more than 10 clinical studies per year revealed that electroacupuncture, TENS, and ICT devices were used on a regular basis every year, and IR equipment, laser equipment, and SSPs were used frequently in 2016 (Figure 4).

### 3.4. Diagnosis and Treatment of Diseases per Type of Medical Device

#### 3.4.1. Diseases Diagnosed by Diagnostic Devices

The 5 most used diagnostic medical devices were used for various diseases. MRI was used to diagnose herniation of the lumbar disc in 16 studies (8.2%), stroke in 12 studies (6.2%), and rotator cuff tear in 5 studies (2.6%). X-ray machines were used for diagnosing scoliosis in 9 studies (5.2%) and pneumonia and herniation of the lumbar disc in 4 studies (2.3%). CT scanners were used to diagnose stroke in 8 studies (5.8%), lung cancer in 5 studies (3.6%), and herniation of the lumbar disc and gastric cancer in 4 studies (2.9%). For diagnosing amyotrophic lateral sclerosis and stroke, EKG was utilized in 4 studies (5.0%). Ultrasound devices were utilized in diagnosing infertility in 4 studies (7.0%), uterine myoma in 3 studies (5.3%), and hepatitis, frozen shoulders, gastrocnemius tear, drug-induced liver injuries, and uterine bleeding in 2 studies (3.5%) (Table 2).

#### 3.4.2. Diseases Treated by Medical Treatment Devices

Investigation of the diseases treated by the 5 most used medical treatment devices revealed that electroacupuncture was used in treating facial palsy in 9 studies (11.0%), stroke in 5 studies (6.1%), and obesity and headaches in 3 studies (4.1%). Transcutaneous electrical nerve stimulation was utilized in treating herniation of the lumbar disc in 11 studies (14.9%), back pain in 4 studies (5.4%), and shoulder pain in 3 studies (4.1%). Interferential current therapy was used for treating herniation of the lumbar disc, in 7 studies (10.6%), while ultrasonography was used in 3 studies (13.0%). Infrared devices were used in 5 different studies (21.7%) to treat facial palsy (Table 3).

## 4. Discussion

The medical devices are important for quality of health service delivery [16]. It is an applied technique that combines various subject fields such as clinical medicine, electricity, electronics, mechanics, and optical science [17]. The industry is part of the health and medical treatment industry, with the aim of improving the quality of life of humans. The current medical device industry in Korea has gone beyond the simple research stage and is transforming itself to a customized industry where replaceable biomaterial and artificial organs are being developed with high technology [18].

When investigating previous research regarding TKM doctors use of medical devices, Kim et al. [19] conducted a survey where 900 TKM doctors were asked what types of medical devices are kept in TKM institutions. It was stated that although medical imaging equipment such as X-ray machines, CT scanners, and MRI scanners were not kept as much in these institutions, the frequency of their use was high. Sakong et al.'s [20] research provided critical views on restrictions imposed on TKM doctors in using medical imaging equipment by investigating cases where TKM doctors were restricted from using medical imaging equipment, how medical imaging equipment was brought into the medical industry, and the academic principles and study curriculum of TKM.

In Taiwan, there are no laws prohibiting the use of modern medical devices by TCM doctors, and some medical devices are available for the ministry of health [7, 15].

In China, there are no laws prohibiting the use of modern medical devices by TCM doctors, and the government does not restrict the use of modern medical devices and western medicine prescriptions by the Chinese government [7, 21]. For this reason, many studies have been conducted combining TCM with medical imaging devices [22–24].

This study shows that diagnostic devices have been used more than treatment devices in TKM clinical studies. When investigating the diagnostic medical devices and treatment devices used in the TKM clinical studies, the statistics of the usage per year show that both types of devices have been used steadily from 2012 to 2016. In the past, TKM doctors made diagnosis based on the traditional observing, smelling, asking, and touching methods and treated their patients with acupuncture, moxibustion, and herbal medicine. Currently, medical devices are used for diagnosis and treatment, indicating advancement in treatment. Initially, even doctors did not use medical devices for diagnosing and treating patients. Based on medical knowledge, objective information driven from utilizing modern medical devices allowed for this change, and it is thought that these types of medical acts will further transform themselves with the development of scientific technology.

The use of MRI scanners, X-ray machines, and CT scanners accounted for 53.7% of the total diagnostic device usage from 2012 to 2016 in TKM clinical studies. However, MRI scanners, CT scanners, X-ray machines, and diagnostic ultrasound machines are medical devices that TKM doctors are not allowed to use by law [25–27], and it is estimated that TKM institutions gather and utilize health information such as image materials taken from CM hospitals or image materials gathered through joint treatment of both TKM and CM doctors.

Clauses 37 and 38 under the Medical Service Act state each of the installment regulations and managing director decisions for MRI, radiography, and CT, and TKM doctors do not have the authority to direct [28]. Clause 1 of the Medical Technicians Act states that leadership over medical technicians can be given to dentists or medical doctors [29]. Furthermore, most of the preceding cases state that the use of modern diagnostic devices by TKM doctors is not permitted. However, there was an exceptional case where the use of 5 different medical devices (e.g., applanation tonometer, autorefractor and keratometer, slit lamp, pure tone audiometer, and automated perimeter) were allowed to a TKM doctor at the constitutional court [30]. The reasons for the denial are thought to be a dual medical system, differences in academic principles, insufficient legal grounds for TKM doctors using medical devices, and insufficient educational institutions. This study suggests that TKM doctors use diagnostic information derived from modern medical devices clinically. In order for the TKM study, which is heavily based on traditional knowledge and experience, to go through the process of digitization and produce evidence, collecting objective information by utilizing medical devices is needed. Although there can be differences in the methods of treatment in TKM and CM, the right to use medical devices utilized for treatment and diagnosing patients should be equally given to both types of doctors. Interferential current therapy and TENS are used for treatment every year. It is thought that they have the effect of relieving muscle tension, making the flow of qi smooth, and stimulating the meridian point to improve the pain. The use of the same treatment device may vary depending on the type of medical occupation. It is necessary to consider this aspect when developing medical devices and licensing items.

Diagnostic medical devices such as MRI scanners, CT scanners, and X-ray machines were widely used in herniation of the lumbar disc. In Korea, the Ministry of Health and Welfare operates a system to designate specialized hospitals that treat specific diseases when the medical institute meets the standards set by Medical Law [31]. A total of 109 specialized hospitals have been designated as of 2018; in the TKM field, 8 TKM hospitals specialized in the spine and 1 TKM hospital specialized in gynecology were designated [31]. It is thought that the results of this study reflect that MRI scanners, CT scanners, and X-ray machines are used for treatment in hospitals that specialize in the spine. Conventional medicine and TKM doctors in these hospitals cooperate in areas of treatment-related research [32].

There are some limitations in this review. First, TKM clinical studies indexed in English databases, including EMBASE and MEDLINE, were not considered in our review. Therefore, it is difficult to see that the clinical trials studies related to TKM included in this paper are representative of the use of medical devices in TKM doctors.

Second, it was impossible to fully explore how TKM doctors utilize medical devices as they have restrictions in using these devices in their practice. To understand how these medical devices are used in actual practice, studies such as surveys or interviews are needed and investigations of medical behaviors of TM doctors in China and Taiwan are required.

Third, most of the clinical studies included in this review were conducted in TKM hospitals, and TKM hospitals make up only 1.9% of the TKM institutions [33]. It is therefore difficult to conclude that this review represents the majority of practices being carried out in TKM institutions.

Although TKM has been responsible for Korean people's health for thousands of years and holding sufficient historical evidence, there is no modern scientific evidence to support TKM. Therefore, this review is valuable because the medical behavior of TKM doctors was investigated for the first time. We cautiously propose that this review contributes to establishing TM policies in countries that practice TM and providing improved medical services of TM to patients.

## 5. Conclusion

We reviewed the use of medical devices by TKM doctors in Korea. It provided an understanding about the recent status and trends of the medical behaviors of TKM doctors related to medical devices. This study suggests that TKM doctors utilize medical devices for diagnosis and treatment of patients. It is necessary to institutionalize considering making changes to the medical acts of TM doctors. Additionally, this information can be utilized as a reference for making TM policy and education.

## Figures and Tables

**Table 1:** Medical devices reported in TKM clinical studies.

Types of Medical Device	Top 10 used Medical Device	Number of papers n (%)
Diagnostic device 942	MRI scanners	194 (20.6)
	X-ray machines	172 (18.3)
	CT scanners	139 (14.8)
	EKG equipment	80 (8.5)
	Ultrasound scanners	57 (6.1)
	DITI equipment	43 (4.6)
	HRV measurement equipment	41 (4.4)
	Body composition analyzer	28 (3.0)
	EMG equipment	21 (2.2)
	Endoscope	19 (2.0)
	Etc.	148

Treatment device 403	Electro acupuncture devices	82 (20.3)
	TENS equipment	74 (18.4)
	ICT equipment	66 (16.4)
	Ultrasound devices	23 (5.7)
	IR equipment	23 (5.7)
	M/W equipment	19 (4.7)
	EST equipment	12 (3.0)
	Laser devices	11 (2.7)
	SSP equipment	11 (2.7)
	Electromagnetic stimulation therapy equipment	6 (1.5)
	Etc.	76

MRI: magnetic resonance imaging; CT: computed tomography; EKG: electrocardiography; DITI: digital infrared thermal imaging; HRV: heart rate variability; EMG: electromyography; TENS: transcutaneous electrical nerve stimulation; ICT: interferential current therapy; IR: infrared rays; M/W: microwave therapy; EST: electrical stimulation therapy; SSP: silver spike point.

**Table 2:** List of diseases diagnosed by diagnostic devices.

Diagnostic Devices	Diseases of Top 3 used	n (%)
MRI scanners	Herniation of lumbar disc	16 (8.2)
	Stroke	12 (6.2)
	Rotator cuff tear	5 (2.6)

X-ray machines	Scoliosis	9 (5.2)
	Pneumonia and herniation of lumbar disc	4 (2.3)

CT scanners	Stroke	8 (5.8)
	Lung cancer	5 (3.6)
	Herniation of lumbar disc and gastric cancer	4 (2.9)

EKG equipment	Amyotrophic lateral sclerosis and stroke	4 (5.0)
	Parkinson disease	2 (2.5)

Ultrasound scanners	Infertility	4 (7.0)
	Uterine myoma	3 (5.3)
	Hepatitis, frozen sholder, gastrocnemius tear, drug-induced liver injury and uterine bleeding	2 (3.5)

MRI: magnetic resonance imaging; CT: computed tomography; EKG: electrocardiography.

**Table 3:** List of diseases treated by treatment devices.

Treatment Devices	Diseases of Top 3 used	n (%)
Electro acupuncture	Facial paralysis	9 (11.0)
	Stroke	5 (6.1)
	Obesity and headache	3 (3.7)

TENS equipment	Herniation of lumbar disc	11 (14.9)
	Back pain	4 (5.4)
	Shoulder pain	3 (4.1)

ICT equipment	Herniation of lumbar disc	7 (10.6)
	Back pain	5 (7.6)
	Neck pain	4 (6.1)

Ultrasound devices	Herniation of lumbar disc	3 (13.0)

IR devices	Facial paralysis	5 (21.7)

TENS: transcutaneous nerve stimulation; ICT: interferential current therapy; IR: infrared rays.
